# A vitamin B_12_ transporter in *Mycobacterium tuberculosis*

**DOI:** 10.1098/rsob.120175

**Published:** 2013-02

**Authors:** Krishnamoorthy Gopinath, Česlovas Venclovas, Thomas R. Ioerger, James C. Sacchettini, John D. McKinney, Valerie Mizrahi, Digby F. Warner

**Affiliations:** 1 MRC/NHLS/UCT Molecular Mycobacteriology Research Unit and DST/NRF Centre of Excellence for Biomedical Tuberculosis Research, Institute of Infectious Disease and Molecular Medicine and Department of Clinical Laboratory Sciences, Faculty of Health Sciences, University of Cape Town, Observatory, Cape Town 7925, South Africa; 2 Laboratory of Bioinformatics, Institute of Biotechnology, Vilnius, Lithuania; 3 Department of Biochemistry and Biophysics, Texas A&M University, College Station, TX, USA; 4 Global Health Institute, Swiss Federal Institute of Technology (EPFL), Lausanne, Switzerland

**Keywords:** tuberculosis, vitamin B_12_, corrinoids, BtuFCD, BacA

## Abstract

Vitamin B_12_-dependent enzymes function in core biochemical pathways in *Mycobacterium tuberculosis*, an obligate pathogen whose metabolism *in vivo* is poorly understood. Although *M. tuberculosis* can access vitamin B_12_ *in vitro*, it is uncertain whether the organism is able to scavenge B_12_ during host infection. This question is crucial to predictions of metabolic function, but its resolution is complicated by the absence in the *M. tuberculosis* genome of a direct homologue of BtuFCD, the only bacterial B_12_ transport system described to date. We applied genome-wide transposon mutagenesis to identify *M. tuberculosis* mutants defective in their ability to use exogenous B_12_. A small proportion of these mapped to *Rv1314c*, identifying the putative PduO-type ATP : co(I)rrinoid adenosyltransferase as essential for B_12_ assimilation. Most notably, however, insertions in *Rv1819c* dominated the mutant pool, revealing an unexpected function in B_12_ acquisition for an ATP-binding cassette (ABC)-type protein previously investigated as the mycobacterial BacA homologue. Moreover, targeted deletion of *Rv1819c* eliminated the ability of *M. tuberculosis* to transport B_12_ and related corrinoids *in vitro*. Our results establish an alternative to the canonical BtuCD-type system for B_12_ uptake in *M. tuberculosis*, and elucidate a role in B_12_ metabolism for an ABC protein implicated in chronic mycobacterial infection.

## 2. Introduction

The genome of *Mycobacterium tuberculosis*, obligate human pathogen and causative agent of tuberculosis, encodes three B_12_-dependent enzymes. Previous work in our laboratory has established that both the methylmalonyl-coenzyme A (CoA) mutase, MutAB [1], and the *metH*-encoded methionine synthase [2] are functional, and require B_12_ for activity. *Mycobacterium tuberculosis* also possesses a predicted pathway for B_12_ biosynthesis [3], but appears not to produce the cofactor *in vitro* [1,2] or in macrophages [4]. Nevertheless, the bacillus can use exogenous vitamin B_12_ and encodes a B_12_-responsive riboswitch that suppresses transcription of the alternative, B_12_-independent methionine synthase, *metE*, in B_12_-replete conditions [2]. These observations imply a role for the cofactor in *M. tuberculosis* pathogenesis. However, it is uncertain whether B_12_ is available during infection, and which mycobacterial genes are required for its uptake and assimilation.

**Figure 1.**
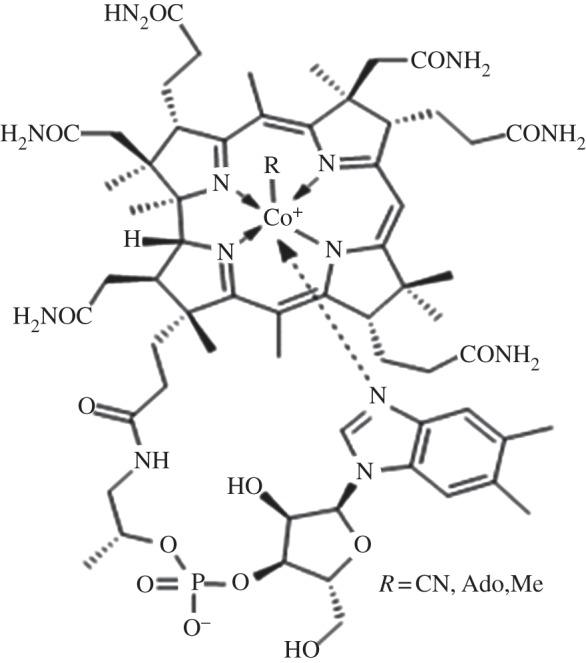
Structure of vitamin B_12_ and B_12_-derived cofactors.

Vitamin B_12_ and B_12_ derivatives are members of the cobalamin group of corrinoid macrocycles [5]. Cobalamins are structurally complex, comprising a defining tetrapyrrole framework with a centrally chelated cobalt ion held in place by a lower axial base, dimethylbenzimidazole and an upper ligand that determines the cofactor form (figure 1). The cyano group in vitamin B_12_ (cyanocobalamin, CNCbl) must be replaced by deoxyadenosine and methyl ligands, respectively, during conversion to the biologically active cofactors: adenosylcobalamin (AdoCbl or coenzyme B_12_), which is required by methylmalonyl-CoA mutase, and methylcobalamin (MeCbl), which serves as an intermediary in the synthesis of methionine from homocysteine and methyltetrahydrofolate [6]. The reactivity of B_12_ cofactors derives from the cobalt-coordinated organic ligands [7] and, together with the size of the cobalamin core, underlies the need for multi-component systems to mediate controlled translocation and delivery of B_12_ across the cell membrane to its target enzyme [8].

Although bioinformatic analyses have predicted alternative vitamin transporters [9], BtuCD–BtuF remains the only confirmed bacterial B_12_ transport system identified to date [10]. The *Escherichia coli* model is the best characterized: a high-affinity corrinoid transporter, BtuB, operates with the TonB–ExbBD complex to traffic B_12_ across the outer membrane into the periplasm [11] where it is captured by the *btuF*-encoded B_12_-binding protein and delivered to the ATP-binding cassette (ABC) importer, BtuCD, which spans the cytoplasmic membrane [12]. *Mycobacterium tuberculosis* is characterized by a notoriously complex cell envelope comprising a cytoplasmic membrane and an external cell wall [13]. However, despite its demonstrated ability to use exogenous B_12_ [2,4], the proteins involved in mycobacterial B_12_ transport and assimilation are unknown: *M. tuberculosis* is included in the small number of B_12_-using bacteria that lack a candidate BtuFCD-type B_12_ transport system [3,9,14] as well as an identifiable homologue of TonB [15].

In this study, we used random mutagenesis to identify genes whose disruption abrogated the ability of *M. tuberculosis* to use exogenous vitamin B_12_ *in vitro*. Our results establish an essential role in B_12_ uptake for Rv1819c, a predicted ABC protein implicated in chronic infection *in vivo* [16], thereby revealing an alternative to the well-characterized BtuCD system for B_12_ transport.

## 3. Material and methods

### 3.1. Bacterial strains and growth conditions

Strains, plasmids and oligonucleotides are described in the electronic supplementary material, table S1. *Mycobacterium tuberculosis* was grown on Middlebrook 7H10 (Difco) supplemented with 0.5 per cent glycerol and Middlebrook OADC enrichment (Difco) or in Middlebrook 7H9 supplemented with 0.2 per cent glycerol, Middlebrook OADC and 0.05 per cent Tween 80 or 0.05 per cent tyloxapol, as required. For propionate utilization experiments, 7H9 broth was supplemented with 0.5 per cent bovine serum albumin fraction V (Sigma), 0.085 per cent NaCl and 0.1 per cent (w/v) sodium propionate, as described [1]. Hygromycin (hyg), kanamycin (kan) and gentamicin (gent) were used at 50, 25 and 2.5 μg ml^−1^, respectively, CNCbl and AdoCbl at 10 µg ml^−1^, (CN)_2_Cbi at 1 µM and 3-nitropropionate (3NP) at 0.1 mM.

### 3.2. Construction of transposon mutant library

A library of transposon (Tn) mutants was constructed in *M. tuberculosis* H37Rv *ΔmetH*, using the MycoMarT7 phage as described [17]. For the primary screen, transductants were plated across multiple 7H10 plates containing 20 µg ml^−1^ kan and 10 µg ml^−1^ CNCbl at a density of 20 000 colony forming units (CFU) per plate. The secondary screen was performed in duplicate in microtitre plate format and, for each Tn mutant, comprised four parallel wells containing 0.1 per cent propionate plus 20 µg ml^−1^ kan as base medium in each well: the first well constituted a growth control and contained only the base medium; in well 2, 10 µg ml^−1^ CNCbl was added to the base medium; in well 3, the base medium was supplemented with 0.1 mM 3NP; and in well 4, 0.1 mM 3NP and 10 µg ml^−1^ CNCbl were added.

### 3.3. Identification of transposon insertion sites

A combination of Tn-linker [18] and rescue cloning [19] strategies was applied to identify Tn insertion sites using the oligonucleotides in the electronic supplementary material, table S1.

### 3.4. Construction of mutant strains of *Mycobacterium tuberculosis*

*Mycobacterium tuberculosis* mutants were constructed using suicide plasmids described in electronic supplementary material, table S1. Genetic complementation used tweety-based vectors [20].

### 3.5. DNA sequencing

*Mycobacterium tuberculosis* genomic DNA was sequenced using an Illumina GenomeAnalyzer II, as described previously [21].

### 3.6. Homology modelling

The initial detection of crystal structures related to Rv1819c was performed using HHsearch [22] and COMA [23]. The Rv1819c model was then generated using a previously described iterative approach [24,25]. Briefly, both the set of structural templates and corresponding alignments were refined until the resulting model stopped improving and the visual inspection revealed no significant flaws.

## 4. Results

### 4.1. A forward genetic screen identifies B_12_ uptake mutants

**Figure 2.**
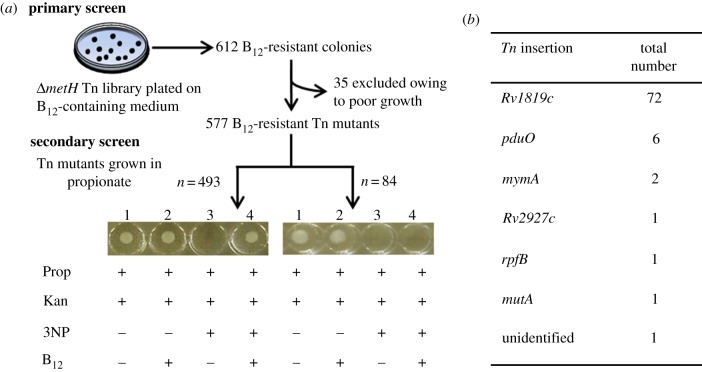
Identification of genes required for B_12_ transport and assimilation. (*a*) Schematic of the screening cascade. The *ΔmetH* Tn library was plated on selective medium containing 10 µg ml^−1^ CNCbl. 612 ‘B_12_-resistant’ clones were isolated and regrown in standard liquid medium, eliminating 35 mutants owing to poor (*n* = 14) or absent (*n* = 21) growth. A secondary screen tested the B_12_ uptake ability of the remaining 577 insertion mutants in a four-well microtitre assay using 0.1% propionate (Prop) plus 20 µg ml^−1^ kanamycin as base medium (well 1) supplemented with 10 µg ml^−1^ CNCbl (well 2), 3NP (well 3) and 3NP plus 10 µg ml^−1^ CNCbl (well 4). A total of 84 mutants failed to grow in well 4, suggesting impaired B_12_ uptake. Each determination was performed in duplicate, and the results confirmed in batch culture. (*b*) Insertion mutants with disrupted B_12_ uptake ability.

We showed previously that deletion of the B_12_-dependent methionine synthase, MetH, renders *M. tuberculosis* sensitive to vitamin B_12_ during growth on solid medium [2]. This phenotype depends on the function of a B_12_ riboswitch that is located immediately upstream of *metE*, the gene encoding an alternative, B_12_-independent methionine synthase in *M. tuberculosis*. In wild-type *M. tuberculosis*, exogenous B_12_ suppresses transcription of *metE* by binding to the riboswitch [2], possibly ensuring efficient B_12_-dependent methionine synthesis by MetH. In the *metH* deletion mutant, however, riboswitch-mediated suppression of *metE* in response to B_12_ effectively results in the complete shutdown of methionine synthase activity, thereby eliminating production of an essential amino acid and so inhibiting bacillary growth [2]. This effect is most profoundly manifest on solid medium, where exposure to 10 µg ml^−1^ CNCbl results in a 3log_10_-fold reduction in viable CFU of *ΔmetH* knockout mutants [2]. Here, we exploited the observed B_12_ sensitivity of *metH* mutants in a genetic screen designed to elucidate a potential B_12_ transport system in *M. tuberculosis* (figure 2). To this end, we constructed an unmarked *metH* knockout of the laboratory strain, *M. tuberculosis* H37RvJO [21] (electronic supplementary material, figure S1*a*) and confirmed that it phenocopied the previously described hygromycin (*hyg*)-marked *ΔmetH* (BB) deletion mutant [2] during growth on B_12_-containing solid medium (see the electronic supplementary material, figure S1*b*). The unmarked *ΔmetH* knockout was used as background strain in which to generate a Tn mutant library using the MycoMarT7 phage [17] that carries a kan resistance marker and inserts randomly at TA dinucleotides [19]. In the primary screen, the library of insertion mutants was plated on solid medium containing kan and CNCbl to enable the identification of genes whose disruption alleviated the growth defect of the *metH* mutant (figure 2*a*). In total, 612 individual clones were isolated, each of which was picked and regrown in standard liquid medium; of these, 35 grew poorly or not at all and were eliminated, leaving 577 ‘B_12_-resistant’ insertion mutants for further analysis.

Previously, in characterizing the *ΔmetH* (BB) mutant, we noted the high frequency at which suppressor mutants arose spontaneously on B_12_-containing solid medium, with single-nucleotide polymorphisms (SNPs) in the B_12_ riboswitch located upstream of *metE* accounting for approximately 10–20 per cent of these [2]. In the current screen, we used dual selection on kan and CNCbl in order to limit the potentially confounding effects of spontaneous riboswitch mutations: according to these criteria, growth on CNCbl plus kan would require successful transduction with the kan-resistant Tn as well as disruption—spontaneous or Tn-mediated—of B_12_-dependent growth inhibition. Nevertheless, we predicted that a significant proportion of B_12_-resistant mutants might contain Tn insertions in the riboswitch motif. So, in order to minimize the impact of disruptions to the B_12_ riboswitch, we applied a secondary screen (figure 2*a*) to determine the capacity of the insertion mutants to assimilate exogenous CNCbl for growth in liquid medium containing propionate in the presence of 3NP, an inhibitor of the key methylcitrate cycle enzyme, isocitrate lyase [26]. Two prior observations informed the design of this screen: (i) the inhibitory effect of genetic (*ΔprpDC*) or chemical (3NP) abrogation of methylcitrate cycle enzymes during growth in liquid medium containing propionate can be alleviated by supplementing the culture with CNCbl, thereby enabling *M. tuberculosis* to use propionate as a carbon source via the methylmalonyl pathway that includes the B_12_-dependent methylmalonyl-CoA mutase, MutAB [1]; (ii) for reasons that are not clear, B_12_-mediated growth inhibition is less effective in liquid versus solid medium—that is, the *ΔmetH* mutant can grow in B_12_-supplemented liquid medium (see the electronic supplementary material, figure S1*c*).

The secondary screen therefore assessed the ability of all 577 Tn mutants to use exogenous CNCbl for growth in liquid medium containing propionate in the presence of 3NP (figure 2*a*). The majority of Tn mutants (*n* = 493) phenocopied the parental *ΔmetH* strain in this assay, and were eliminated as candidate B_12_ uptake mutants. In contrast, the remaining 84 Tn mutants were unable to grow in well 4, suggesting impaired ability to use exogenous B_12_ for methylmalonyl pathway-dependent propionate catabolism. To verify these results, 43 of the 84 mutants were selected at random for phenotypic confirmation of disrupted B_12_ uptake in batch culture (data not shown) and on B_12_-containing solid medium (see the electronic supplementary material, figure S2*a*).

### 4.2. Disruption of *Rv1819c* eliminates the ability of *Mycobacterium tuberculosis* to use exogenous B_12_ *in vitro*

**Figure 3.**
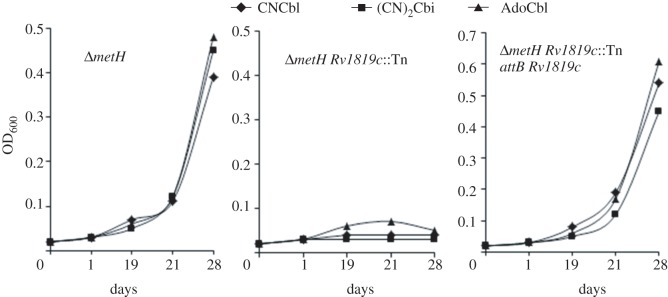
Disruption of *Rv1819c* eliminates the ability of *M. tuberculosis* *ΔmetH* to use corrinoids for growth in 0.1% propionate-containing 3NP. Data are from a representative experiment performed in duplicate.

Insertions in *Rv1819c* accounted for 72 of the 84 Tn mutants (figure 2*b*; electronic supplementary material, figure S2*a*–*c*) and, moreover, mapped throughout the 1920 bp gene (electronic supplementary material, figure S2*d*). This result strongly suggested a role in B_12_ uptake for a predicted ABC transport protein previously identified as the putative *M. tuberculosis* homologue of BacA [16,27], a protein of cryptic function implicated in chronic infection in multiple host–pathogen models [25]. In their study of *M. tuberculosis* Rv1819c, Domenech *et al*. [16] constructed a deletion mutant by allelic exchange mutagenesis (see the electronic supplementary material, figure S2*b*). We assessed the ability of this mutant—referred to as *ΔbacA*::*hyg* by Domenech *et al*. [16]—to use exogenous B_12_ for MutAB-dependent growth in propionate (electronic supplementary material, figure S3*a*). Consistent with the inferred role of *Rv1819c* in B_12_ uptake, the *ΔbacA*::*hyg* strain grew very poorly in propionate plus 3NP supplemented with B_12_, reproducing the phenotype of the *ΔmetH Rv1819c*::Tn mutants (figure 3). By contrast, the complemented derivative carrying a full-length copy of *Rv1819c* at the *attB* site, referred to as *ΔbacA*::pKLMt5 in the original study [16], was able to use B_12_ for growth (see the electronic supplementary material, figure S3*a*). Similarly, integration of full-length *Rv1819c* at *attB* restored the B_12_-sensitive phenotype of a randomly selected *ΔmetH Rv1819c*::Tn mutant during growth on solid medium supplemented with CNCbl (see the electronic supplementary material, figure S3*b*), and reversed the inability of the same mutant to use B_12_ for growth in propionate-containing liquid medium supplemented with 3NP (figure 3), confirming the essentiality of Rv1819c in this assay.

It was noticeable in the propionate utilization experiment (see the electronic supplementary material, figure S3*a*) that the *ΔbacA*::*hyg* mutant started to replicate after two to three weeks of apparent growth arrest, possibly indicating the emergence of suppressor mutants. To circumvent this complication, we deleted the *prpDC* locus [28] in this strain, thereby negating the need to use 3NP to eliminate methylcitrate pathway function [1]. In contrast to the single *prpDC* deletion mutant, the double *ΔbacA*::*hyg* *ΔprpDC* knockout exhibited no growth at all in propionate over the 28-day time course (see the electronic supplementary material, figure S3*c*), even when supplemented with CNCbl, strongly suggesting that Rv1819c is required for the assimilation of exogenous B_12_ to enable methylmalonyl-CoA pathway function.

### 4.3. Spontaneous B_12_-resistant mutants carrying non-synonymous single-nucleotide polymorphisms in *Rv1819c*

We reported previously that SNPs in the *metE*-associated B_12_ riboswitch accounted for 10–20 per cent of all B_12_-resistant mutants isolated after plating the *ΔmetH* (BB) knockout on medium containing CNCbl, whereas the remaining B_12_-resistance mutations were unknown [2]. To investigate the possibility that mutations in *Rv1819c* might account for B_12_ resistance in those clones lacking riboswitch mutations, we plated the *ΔmetH* (BB) strain on medium containing CNCbl and sequenced the riboswitch region and *Rv1819c* locus in 10 spontaneous B_12_-resistant mutants. Consistent with previous results [2], two isolates carried independent mutations in the highly conserved B12-box motif within the *metE* riboswitch [29], namely C → T transversions at positions −155 and −163 relative to the *metE* start codon, respectively. Notably, four other B_12_-resistant mutants had wild-type riboswitch sequences, but contained non-synonymous SNPs in *Rv1819c* (see the electronic supplementary material, table S2), supporting the inferred role of Rv1819c in B_12_ uptake. To eliminate the possibility that an additional, unidentified mutation (or mutations) might account for the observed phenotype, we sequenced the genome of a representative *Rv1819c* point mutant, SP09 (see the electronic supplementary material, table S2). The parental, B_12_-sensitive strain, *ΔmetH* (BB), was differentiated from the laboratory strain, H37RvJO [21], only in the targeted deletion of *metH* sequence. Moreover, the *Rv1819c* mutation constituted the sole polymorphism separating SP09 from its *ΔmetH* (BB) parent and, importantly, complementation with wild-type *Rv1819c* at the *attB* locus restored B_12_ sensitivity to both SP09 and SP18 (see the electronic supplementary material, figure S4).

In the primary Tn screen (figure 2*a*), ‘B_12_-resistant’ mutants had been selected on kan and CNCbl in order to limit the potentially confounding effects of spontaneous riboswitch mutations. To verify the utility of this approach, we analysed the insertion sites in a random selection of 20 of the 493 *ΔmetH* Tn mutants subsequently eliminated in the secondary screen owing to their inability to use exogenous B_12_ for growth in propionate. All 20 mutants contained insertions in the B_12_ riboswitch region directly upstream of *metE* (data not shown), confirming that disrupted riboswitch function represents a major mechanism for loss of B_12_ regulation in strains which carry an intact *Rv1819c* gene.

### 4.4. Rv1819c is essential for corrinoid transport in *Mycobacterium tuberculosis*

*Mycobacterium tuberculosis* is predicted to encode a complete pathway for B_12_ biosynthesis, including enzymes required for the conversion of the B_12_ precursor, cobinamide, to AdoCbl through the addition of dimethylbenzimidazole and deoxyadenosine ligands [3]. The *E. coli* corrinoid transporter, BtuFCD, mediates uptake of cobinamide as well as CNCbl and AdoCbl [30], suggesting that Rv1819c might fulfil a corresponding role in *M. tuberculosis*. In support of this idea, cobinamide—provided as the dicyanide salt, (CN)_2_Cbi—was unable to complement the growth defect of *ΔmetH Rv1819c*::Tn mutants in propionate in the presence of 3NP, mimicking similar observations with AdoCbl and CNCbl (figure 3). Insertions in *Rv1819c* also alleviated the growth inhibitory effect of AdoCbl, CNCbl and (CN)_2_Cbi on the *metH* knockout mutant on solid medium, a phenotype that was reversed upon complementation with wild-type *Rv1819c* (see the electronic supplementary material, figure S5). In combination, these results confirmed the essentiality of Rv1819c for corrinoid transport in *M. tuberculosis*.

### 4.5. Impaired vitamin B_12_ uptake in spontaneous bleomycin-resistant *Rv1819c* mutants

**Figure 4.**
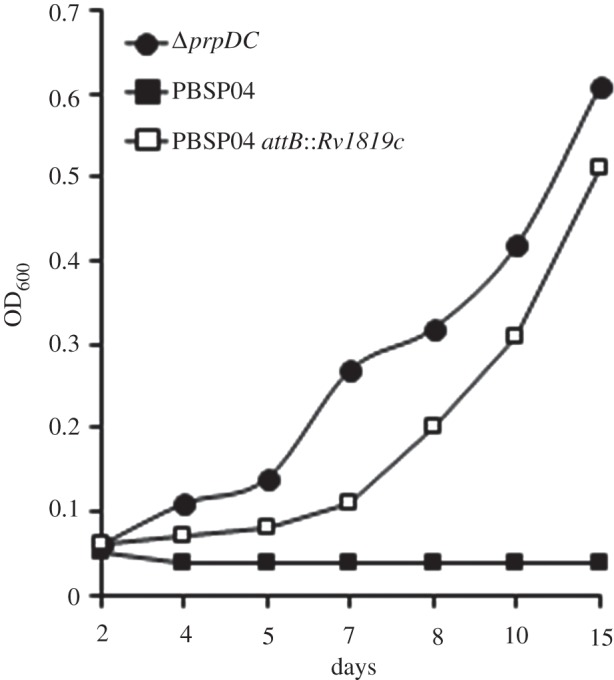
Impaired B_12_ uptake in a spontaneous bleomycin-resistant (Bleo^R^) *prpDC* mutant, PBSP04, carrying a SNP in *Rv1819c* (see the electronic supplementary material, table S2). Strains were grown in 0.1% propionate supplemented with 3NP and CNCbl. Data are from a representative experiment performed in duplicate.

Domenech *et al*. [16] showed that deletion of *Rv1819c* decreased the susceptibility of *M. tuberculosis* to the glycopeptide antibiotic, bleomycin, a phenotype commonly associated with BacA function [31–33]. We determined the minimum inhibitory concentration (MIC) of bleomycin against a selected *Rv1819c*::*Tn* mutant (electronic supplementary material, figure S6*a*) as well as the spontaneous B_12_-resistant mutants, SP09 and SP18 (see the electronic supplementary material, figure S6*b*), and observed values comparable to that reported for *ΔbacA*::*hyg* [16]. To explore further the overlap between B_12_ uptake and bleomycin susceptibility, we isolated spontaneous bleomycin-resistant (Bleo^R^) mutants in two different genetic backgrounds, *ΔprpDC* and the unmarked *metH* knockout, by plating the strains on solid medium containing 3 µg ml^−1^ bleomycin (10 × MIC). Five Bleo^R^ mutants each of the *ΔprpDC* and *ΔmetH* knockouts were selected at random*,* and shown to be defective in their ability to use B_12_ for MutAB-dependent growth in propionate (see the electronic supplementary material, figure S7*a*). Moreover, the spontaneous Bleo^R^ mutants of *ΔmetH* were resistant to CNCbl during growth on solid medium (see the electronic supplementary material, figure S7*b*). All five Bleo^R^ mutants derived from the *ΔprpDC* strain carried nonsense mutations in *Rv1819c*, whereas missense mutations in *Rv1819c* were identified in four of five spontaneous Bleo^R^ *ΔmetH* mutants (see the electronic supplementary material, table S2). Moreover, complementation with full-length *Rv1819c* reversed the inability of the spontaneous *Rv1819c* point mutants of *ΔprpDC* to catabolize propionate in liquid medium supplemented with CNCbl (figure 4), and restored the bleomycin susceptibility of SP09 to wild-type levels (see the electronic supplementary material, figure S6*c*).

### 4.6. *Rv1819c* encodes an ATP-binding cassette-type transporter

Rv1819c was previously included in a group of ‘BacA-related’ proteins identified on the basis of their similarity to the highly conserved BacA and SbmA proteins of *Sinorhizobium* and *E. coli*, respectively [27]. Unlike BacA/SbmA orthologues, however, which are predicted to require an interaction with a separate cytoplasmic protein for function, *Rv1819c* encodes both transmembrane (TMD) and nucleotide-binding (NBD) domains of an ABC transport protein on a single polypeptide. Sequence similarity analyses using only the TMD located *M. tuberculosis* Rv1819c in a cluster distinct from BacA/SbmA (see the electronic supplementary material, figure S8*a*). Moreover, these analyses indicated that Rv1819c was more closely related to ABC proteins other than BacA/SbmA in both *E. coli* and *Sinorhizobium*, namely YddA [34] and ExsE [35], respectively. The Rv1819c NBD similarly identified YddA and ExsE as close homologues in an equivalent similarity search (see the electronic supplementary material, figure S8*b*), together with the recently described human ABC-type B_12_ transporter, ABCD4 [36].

**Figure 5.**
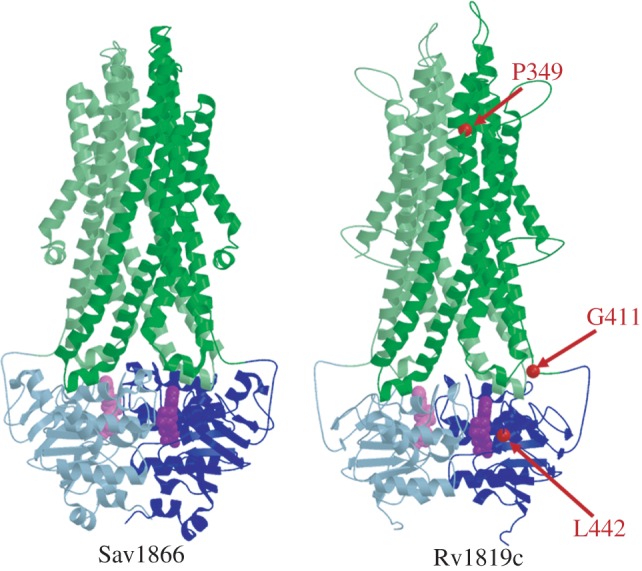
*Rv1819c* encodes an ABC-type transporter. Computational model of *M. tuberculosis* Rv1819c (amino acid residues 66–639) compared with the x-ray structure of *Staphylococcus aureus* Sav1866 (PDB code: 2HYD) [37]. The model is based on the crystal structures of Sav1866 and the ABC lipid flippase, MsbA, from *Salmonella typhimurium* (PDB code: 3B60) [38], both of which contain transmembrane- (green) and nucleotide-binding (blue) domains fused into a single polypeptide chain that interacts to form a homodimer in the active protein. The two subunits in both structures are denoted by the different colour intensities. ADP molecules bound to each subunit are shown in purple and light purple, respectively. Residues substituted in spontaneous *Rv1819c* mutants are indicated with red arrows.

We built a homology model of Rv1819c based on the crystal structures of two polyspecific ABC exporters, *Staphylococcus aureus* Sav1866 [37] and *Salmonella typhimurium* MsbA [38]. Consistent with known ABC protein architecture [39], Rv1819c is predicted to form a homodimer (figure 5), with each subunit comprising an N-terminal TMD fused to a highly conserved NBD that features all the motifs characteristic of functional ABC transporters (see the electronic supplementary material, figure S9*a*). Unlike Sav1866 and MsbA, though, the TMD domain of Rv1819c possesses an extra N-terminal region which is predicted to contain an additional transmembrane helix (see the electronic supplementary material, figure S9*b*). Proteomic analyses in the closely related *M. bovis* BCG suggest that this region is present in the mature protein [40], and therefore is not a signal peptide. However, in the absence of a close structural template containing seven transmembrane helices, we omitted the first 65 N-terminal residues in building the Rv1819c model. The predicted structure nevertheless provides a useful framework for the interpretation of experimental data. Notably, all three SNPs which resulted in substituted amino acids in the spontaneous B_12_-resistant and Bleo^R^ mutants (see the electronic supplementary material, table S2) affect residues located in conserved regions of Rv1819c (figure 5). While the structural consequences of the P349T and G411D mutations require further investigation, L442S affects a conserved position in the putative nucleotide-binding pocket formed by two interacting ABC domains. In Sav1866, the corresponding residue, Ile356, makes a van der Waals contact with the sugar moiety of the bound ADP [37], and so supports the inferred association between a distorted pocket and crippled protein function.

### 4.7. A PduO-type adenosyltransferase is required for assimilation of vitamin B_12_

**Figure 6.**
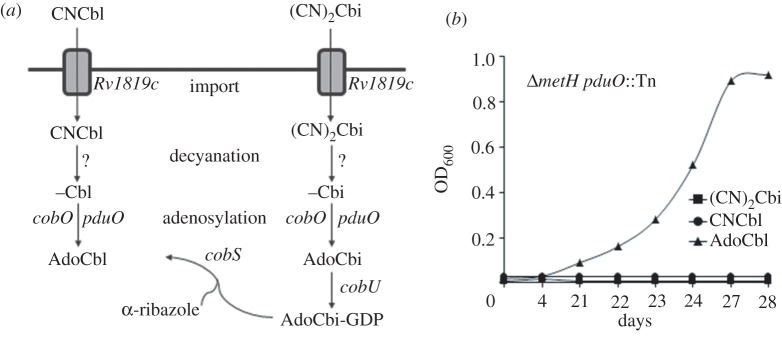
PduO is essential for B_12_ salvage and assimilation. (*a*) Predicted steps in late-stage AdoCbl biosynthesis and salvage in *M. tuberculosis*. Cobinamide is provided *in vitro* as a dicyanide salt, (CN)_2_Cbi*.* (*b*) The *ΔmetH pduO*::Tn mutant cannot use CNCbl or (CN)_2_Cbi for growth in 0.1% propionate-containing 3NP. Data are from a representative experiment performed in duplicate.

CNCbl must be adenosylated to generate the active cofactor, AdoCbl [41] (figure 6*a*). The genome of *M. tuberculosis* is predicted to encode both CobO (*Rv2849c*) and PduO (*Rv1314c*) ATP:co(I)rrinoid adenosyltransferases [3], non-homologous enzymes that catalyse this reaction in other bacteria [42,43]. It was notable, therefore, that six putative B_12_ uptake mutants (figure 2) contained Tn insertions in *pduO* (see the electronic supplementary material, figure S2*d*), because this suggested that an impaired ability to convert exogenous CNCbl to the cofactor form could confer B_12_ resistance in the primary screen, as well as eliminate the ability of *M. tuberculosis* to use B_12_ for growth in propionate. To confirm the role of PduO-dependent adenosylation in these phenotypes, we evaluated the abilities of the *ΔmetH pduO*::Tn mutants to assimilate different corrinoids for growth in propionate in the presence of 3NP (figure 6*b*). The mutants were unable to use either cyano form, CNCbl or (CN)_2_Cbi, both of which are adenosylated in the biosynthetic pathway to AdoCbl (figure 6*a*). In contrast, supplementation with AdoCbl itself restored growth in this assay (figure 6*b*), suggesting bypass of PduO function. In combination, these observations implicate PduO as sole active adenosyltransferase in *M. tuberculosis* during growth *in vitro*.

## 5. Discussion

Our results identify Rv1819c as sole corrinoid transporter in *M. tuberculosis* under standard *in vitro* conditions and, moreover, establish the capacity of the organism to scavenge corrinoids. The association of Rv1819c with B_12_ uptake is unexpected, particularly given previous studies suggesting that Rv1819c might function in ATP-dependent peptide transport [16,44]. Moreover, the properties enabling polyspecific translocation of compounds such as bleomycin, B_12_ and antimicrobial peptides which lack obvious structural similarity remain unclear [45]. In Gram-positive organisms, ABC-mediated importers function together with a high-affinity substrate-binding protein (SBP) that is anchored to the extracytoplasmic membrane [46]. Although *M. tuberculosis* possesses in excess of 30 ABC transporters, as well as 15 putative SBPs [47], we failed to identify a candidate B_12_-binding protein, raising the possibility that Rv1819c-mediated B_12_ import occurs in the absence of a specific SBP or that multiple proteins fulfil this role [48]. In most bacteria, the components of the ABC transporters involved in the uptake of ferric siderophores, haem and vitamin B_12_ are closely related [49]. However, our screen identified Rv1819c as sole transport candidate, excluding the possibility that other ABC proteins might perform overlapping functions in mycobacterial B_12_ transport, at least under *in vitro* conditions. Instead, in associating Rv1819c with B_12_ uptake, our results add to the expanding complement of atypical mycobacterial nutrient acquisition systems. For example, a novel pathway was recently elucidated that enables the scavenging of haem [50]—a tetrapyrrole which, like B_12_, is derived from δ-aminolaevulinic acid via a uroporphyrinogen III intermediate [5]. In that system, uptake is mediated by the combined activity of a haem-binding protein and the MmpL family members MmpL3 and MmpL11—predicted RND-type efflux pumps which have been associated with multiple cellular functions [51]. In addition to haem import, recent evidence suggests that MmpL3 fulfils an essential role in exporting trehalose monomycolate across the cell membrane for incorporation into cell wall mycolic acids [52,53], and it has also been implicated in the susceptibility of *M. tuberculosis* to diverse small molecules [54,55]. It is tempting, therefore, to consider the analogy with Rv1819c—itself a predicted export protein that has now been implicated in the uptake of antimicrobial peptides [16,44] and vitamin B_12_, and might also play a role in cell wall biogenesis [16].

Rv1819c has been extensively investigated as *M. tuberculosis* BacA [16,44]. Unlike BacA/SbmA orthologues, however, deletion of *Rv1819c* does not render *M. tuberculosis* hypersusceptible to other antimicrobial drugs and cell disrupting agents [16]. Moreover, our structural model of *M. tuberculosis* Rv1819c predicts an ABC transporter comprising both TMD and NBD within a single polypeptide. This distinguishes the mycobacterial protein from BacA proteins in *Brucella* and other intracellular pathogens [32] that contain the TMD only and, importantly, is supported by sequence analyses that situate Rv1819c in a separate cluster from the BacA subfamily even when based on TMD sequence alone. The *M. tuberculosis* protein also differs from BacA proteins in its potential role in pathogenesis. While the essentiality of the mycobacterial protein for the maintenance of chronic infection *in vivo* [16] is reminiscent of BacA-like phenotype, closer inspection of the comparative *in vivo* infection dynamics of different ‘*bacA*’ mutants suggests divergent function: for example, in contrast to the *Brucella* and *Sinorhizobium* deletion mutants [27,32], the *M. tuberculosis Rv1819c* knockout is not impaired in its ability to establish an infection [16]. It is tempting, therefore, to consider the virulence defect of the *Rv1819c* deletion mutant in the light of recent studies describing the accumulation during chronic infection of cholesterol-rich lipid bodies inside foamy macrophages and their infecting bacilli [56,57]. That is, Rv1819c might function to ensure adequate supply of host-derived corrinoids for the B_12_-dependent utilization of propionate derived from cholesterol catabolism, a possibility that requires further investigation.

Although designed to detect a putative vitamin B_12_ transporter, our screen also established the essentiality of the PduO-type adenosyltransferase for the assimilation of exogenous corrinoids. The *M. tuberculosis* genome contains both *pduO* and *cobO* adenosyltransferases; therefore, the inferred inactivity of the alternative enzyme *in vitro* might indicate functional adaptation of CobO to *de novo* B_12_ biosynthesis [3], or to specific environmental conditions, including anaerobiosis [41]. Intracellular trafficking of B_12_ in humans requires the sequential activity of multiple proteins which fulfil dual roles as molecular chaperones and in the enzymatic modification of the cofactor [58]. For example, MMACHC catalyses the reductive decyanation of CNCbl [59] while mediating LMBD1-dependent [60] transfer from the lysosome into the cytoplasm. Recent evidence further suggests that this process is facilitated by the interaction of LMBD1 with ABCD4 [36]—an ABC transporter and homologue of Rv1819c (see the electronic supplementary material, figure S8). In a subsequent step, the ATP:corrinoid adenosyltransferase attaches the axial ligand and ensures delivery of the resulting AdoCbl cofactor across the mitochondrial membrane to its target enzyme, methylmalonyl-CoA mutase [61]. Given that Tn-mediated disruption of *pduO* alleviated the B_12_ sensitivity of the *metH* mutant in the primary screen, it is tempting to speculate that PduO might function not merely in enzymatic conversion of exogenous corrinoids, but also in delivery of the active cofactor into the cytoplasm. Our current model for the translocation of B_12_ across the mycobacterial cell wall into the cytoplasm therefore proposes the sequential activity of the ABC transporter, Rv1819c and the PduO-type adenosyltransferase (figure 6*a*).

Our Tn screen also identified four low-frequency insertions associated with compromised B_12_ uptake (figure 2*b*). Targeted sequencing of *Rv1819c* and the *metE* riboswitch in these strains excluded spontaneous mutations as the underlying cause of the observed B_12_ phenotypes. A single mutant carried an insertion in *rpfB*, which encodes a resuscitation-promoting factor. To explore this result further, we retested *ΔmetH rpfB*::Tn in parallel with an *rpfB* deletion mutant of H37Rv, constructed previously [62]. Although the Tn mutant was not able to use exogenous CNCbl for growth in propionate-containing medium, the *ΔrpfB* knockout strain phenocopied wild-type H37Rv in this assay (data not shown), thereby excluding a role for RpfB in B_12_ uptake. It is possible that *rpfB*::Tn possesses an additional, unidentified polymorphism, affecting B_12_ assimilation; alternatively, polar effects on the downstream gene, *ksgA*, encoding dimethyladenosine transferase, might contribute to the observed phenotype [63], a possibility under investigation. Two additional Tn insertions mapped to *mymA*, encoding a putative flavin-dependent monooxygenase. The predicted role of MymA in the maintenance of cell wall ultrastructure [64] suggests that compromised B_12_ uptake in these mutants might be non-specific; however, this requires further investigation, and is complicated by the fact that *mymA* is the first gene in a seven-gene operon [65]. We also isolated a *mutA*::Tn mutant, whose inability to use propionate for growth in B_12_-containing medium is consistent with impaired methylmalonyl-CoA mutase function. The basis for the B_12_ resistance of this mutant in the primary screen is unclear, however, and probably also the result of an additional spontaneous mutation. The final Tn insertion mapped to *Rv2927c*, a gene which previous saturation mutagenesis studies have predicted as essential for growth of *M. tuberculosis in vitro* [66,67]. Although the function of Rv2927c is unknown, it has been proposed to operate as part of the cell division machinery [68]. It seems probable that, like the *mymA*::Tn mutants, the failure of *Rv2927c*::Tn to assimilate B_12_ is non-specific. However, given the inferred requirement for PduO-dependent adenosylation in the assimilation of exogenous B_12_, the prediction that Rv2927c might function in *de novo* adenosine nucleotide biosynthesis [69] is intriguing, and the subject of current investigation.

## 6. Acknowledgements

This work was financially supported by a Swiss–South African Joint Research Programme grant (to V.M. and J.D.M), and by grants from the South African Medical Research Council (to V.M.), the National Research Foundation (to V.M.) and the Howard Hughes Medical Institute (International Research Scholar's grants to V.M. and Č.V.). We thank Eric Rubin and Chris Sassetti for providing the MycoMar phage and for valuable advice, Clif Barry and Helena Boshoff for insightful discussions and for the *ΔbacA*::*hyg* knockout mutant, and Candice Soares De Melo for technical assistance.

## Supplementary Material

Gopinath et al_Supplementary Information_Revised
