# Supplementary material for: A vitamin B12 transporter in Mycobacterium tuberculosis
Source: Open Biol. 2013 Feb;3(2):120175. doi: 10.1098/rsob.120175 (PMC3603451; doi:10.1098/rsob.120175)
Supplement: Gopinath et al_Supplementary Information_Revised [file rsob120175-s1.pdf]

**SUPPLEMENTARY INFORMATION**

# A vitamin $B_{12}$ transporter in *Mycobacterium tuberculosis*

Krishnamoorthy Gopinath[1], Česlovas Venclovas[2], Thomas R. Ioerger[3], James C. Sacchettini[3], John D. McKinney[4], Valerie Mizrahi[1] and Digby F. Warner[1]

[1]MRC/NHLS/UCT Molecular Mycobacteriology Research Unit and DST/NRF Centre of Excellence for Biomedical Tuberculosis Research, Institute of Infectious Disease and Molecular Medicine and Department of Clinical Laboratory Sciences, Faculty of Health Sciences, University of Cape Town, Observatory, Cape Town 7925, South Africa; [2]Laboratory of Bioinformatics, Institute of Biotechnology, Vilnius, Lithuania; [3]Department of Biochemistry and Biophysics, Texas A&M University, College Station, Texas, USA; [4]Global Health Institute, Swiss Federal Institute of Technology (EPFL), Lausanne, Switzerland.

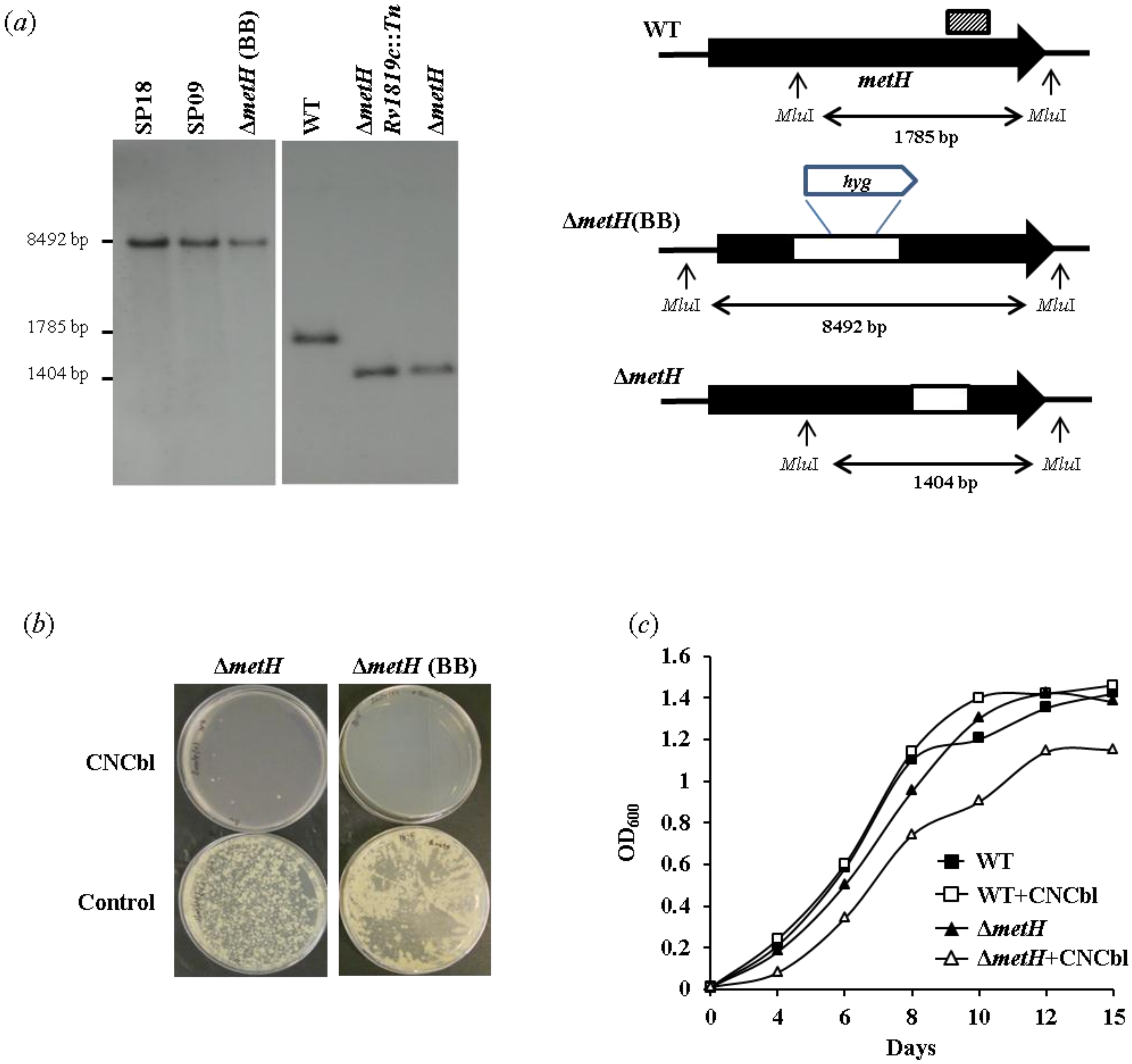


**Figure S1.** Disruption of *metH* renders *M. tuberculosis* sensitive to exogenous vitamin $B_{12}$. (*a*) Genotypic characterization of Δ*metH* parental strains and derivative mutants. Genomic DNA was digested with *Mlu*I and probed with a 644 bp *Mlu*I-*Bgl*II fragment internal to wildtype *metH* (metHp, represented by a hatched box). Restriction maps are illustrated schematically in the line drawings adjacent to the Southern blot (not to scale). The Δ*metH* (BB) allele eliminates 1417 bp *metH* coding sequence including an *Mlu*I site (WT, wildtype H37Rv). The strains analyzed here are the *hyg*-marked *metH* deletion mutant, Δ*metH* (BB), which was described previously (1); spontaneous $B_{12}$-resistant mutants of Δ*metH* (BB), SP09 and SP18 (Table S1); the unmarked Δ*metH* mutant used to construct the Tn library; a representative Δ*metH Rv1819c*::Tn insertion mutant; and wildtype H37RvJO (2). (*b*) Effect of exogenous vitamin $B_{12}$ on growth of Δ*metH* mutants. Cells were incubated on solid 7H10 medium containing 10 µg/ml CNCbl. Representative plates are shown from two independent experiments performed in duplicate. (*c*) The Δ*metH* mutant exhibits a minor growth defect relative to WT *M. tuberculosis* H37Rv in standard liquid medium supplemented with 10 µg/ml CNCbl.

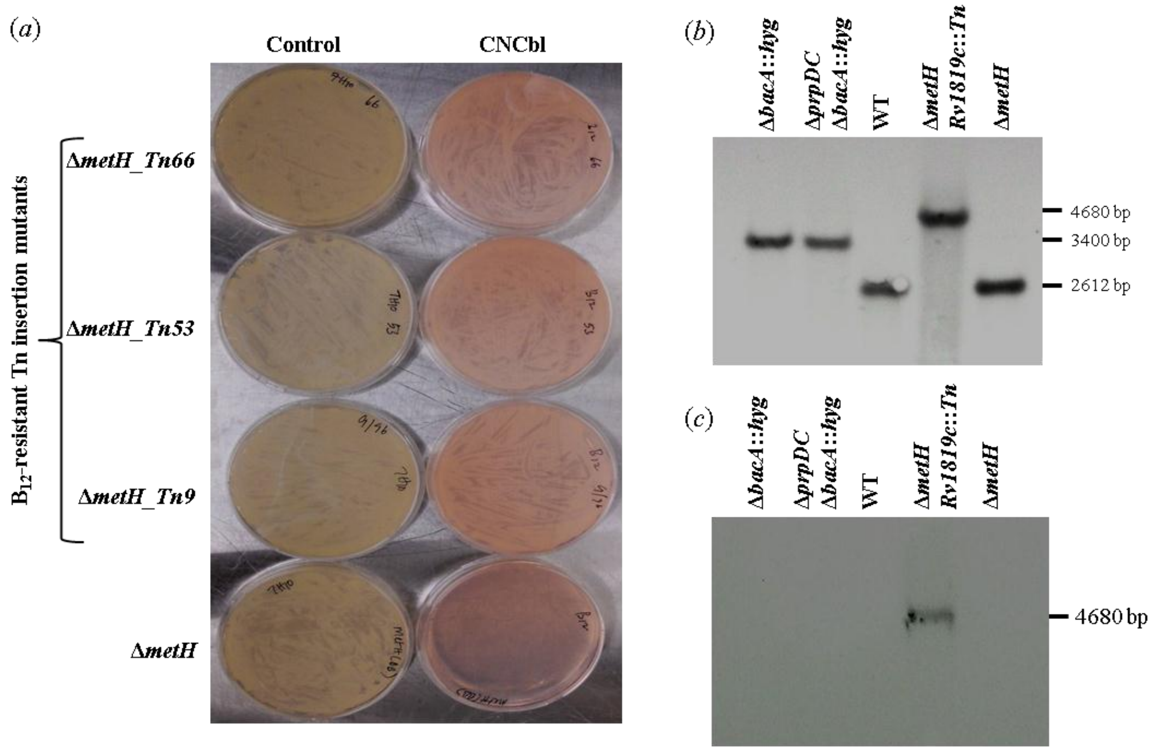


(d)

| Gene | Length (bp) | Number of -TA- sites | Total insertions | Insertions mapped* |
|---|---|---|---|---|
| *Rv1819c (bacA)* | 1920 | 34 | 72 | 173 261 471 580 863 931 971 980 1163 1306 1436 1502 1538 1732 1919 |
| *Rv1314c (pduO)* | 581 | 16 | 6 | 106 286 |
| *Rv3083 (mymA)* | 1488 | 26 | 2 | 380 |
| *Rv2927c* | 738 | 13 | 1 | 216 |
| *Rv1009 (rpfB)* | 1089 | 12 | 1 | 187 |
| *Rv1492 (mutA)* | 1848 | 17 | 1 | 177 |

*For *Rv1819c* and *Rv1314c*, Tn insertion sites were mapped by sequencing 25/72 and 2/6 clones, respectively; the remaining insertions were confirmed by PCR. Values in the red arrows denote the number of insertion mutants mapping to a specific –TA– dinucleotide whose position within the gene is indicated in black text.

**Figure S2.** (*a*) Effect of exogenous vitamin $B_{12}$ on growth of Δ*metH* Tn insertion mutants. Cells were incubated on solid 7H10 medium containing 10 µg/ml CNCbl. Representative plates are shown from independent experiments performed in duplicate. (*b*) Genotypic analysis of *Rv1819c* mutants. Genomic DNA was digested with *Sph*I and hybridized with a *Rv1819c*-specific probe. (*c*) The blot in (*b*) was washed and re-hybridized with a MycoMar-1-specific probe to confirm a representative *metH Rv1819c*::Tn mutant. (*d*) Tn insertion sites identified in 83 of 84 putative "B12 uptake" defective mutants of Δ*metH*.

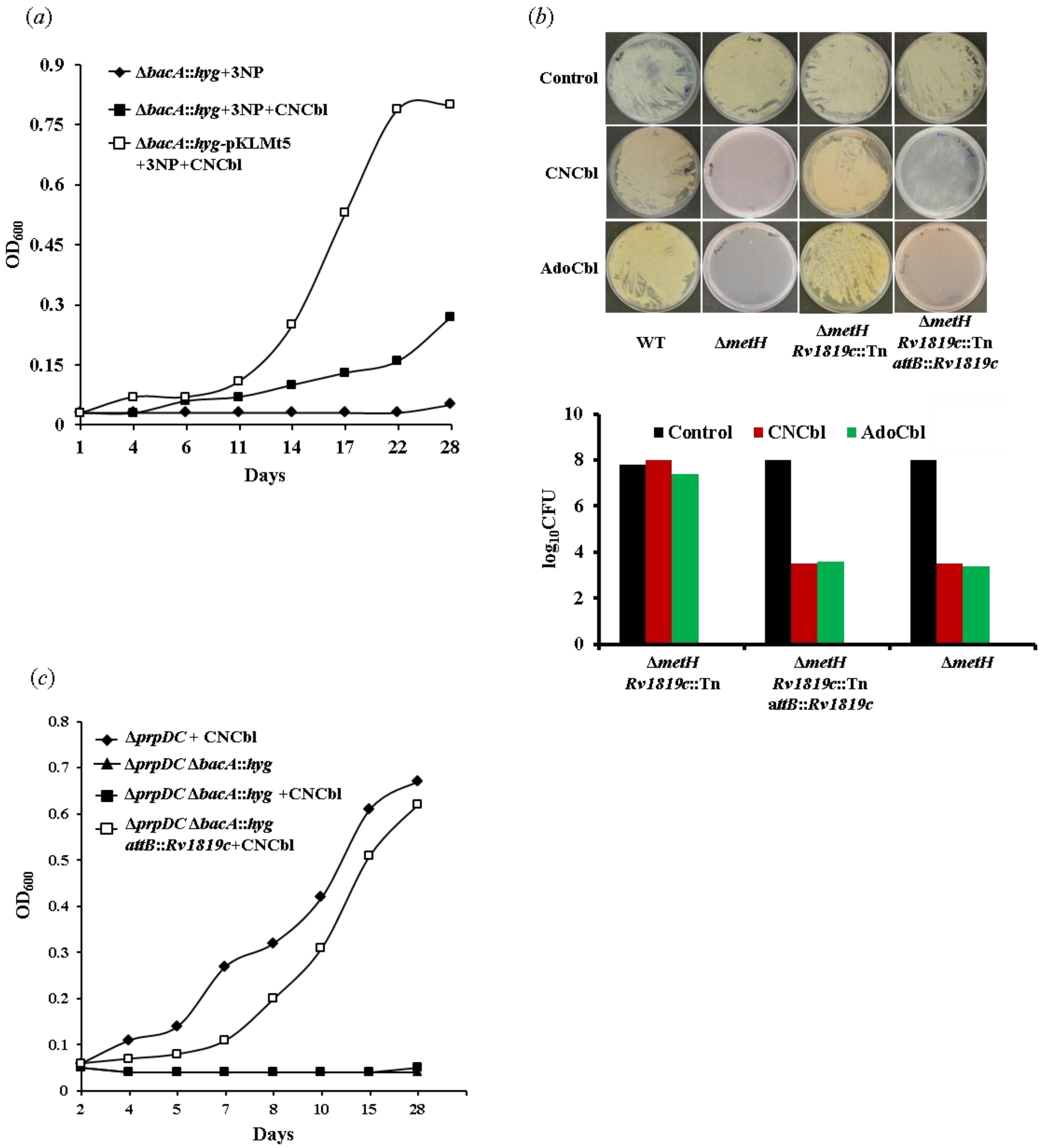


**Figure S3.** Disruption of *Rv1819c* eliminates uptake of vitamin $B_{12}$ in *M. tuberculosis*. (*a*) The Δ*bacA*::*hyg* deletion mutant (3) is unable to utilize $B_{12}$ for growth in 0.1% propionate containing 3NP. Data are from a representative experiment performed in duplicate. (*b*) Effect of exogenous vitamin $B_{12}$ on growth of Δ*metH* parental strain and a representative Δ*metH Rv1819c*::Tn mutant. Cells were incubated on solid 7H10 medium containing CNCbl or AdoCbl at 10 µg/ml, and CFUs scored after 4 weeks. Representative plates are shown from two independent experiments performed in duplicate, and CFU counts plotted in the adjacent bar graph. (*c*) Deletion of *Rv1819c* eliminates the ability of a methylcitrate cycle-deficient Δ*prpDC* mutant to utilize $B_{12}$ for growth in propionate. Data are from a representative experiment performed in duplicate.

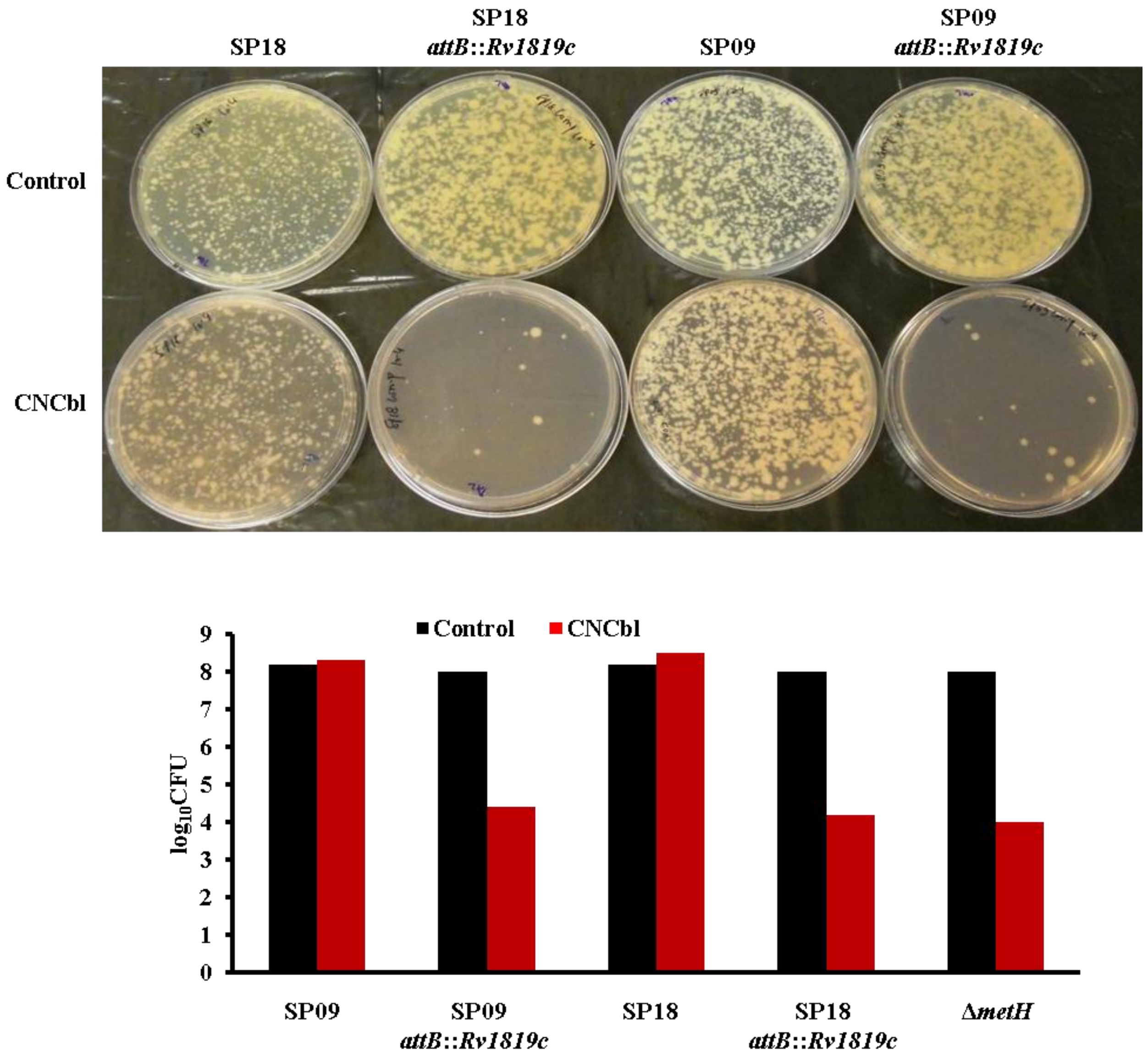


**Figure S4.** SNPs in *Rv1819c* alleviate $B_{12}$ sensitivity in spontaneous $B_{12}$-resistant mutants of Δ*metH*. Cells were incubated on solid 7H10 medium containing 10 µg/ml CNCbl and CFUs were scored after 4 weeks. Representative plates are shown from two independent experiments performed in duplicate, with CFU counts plotted below.

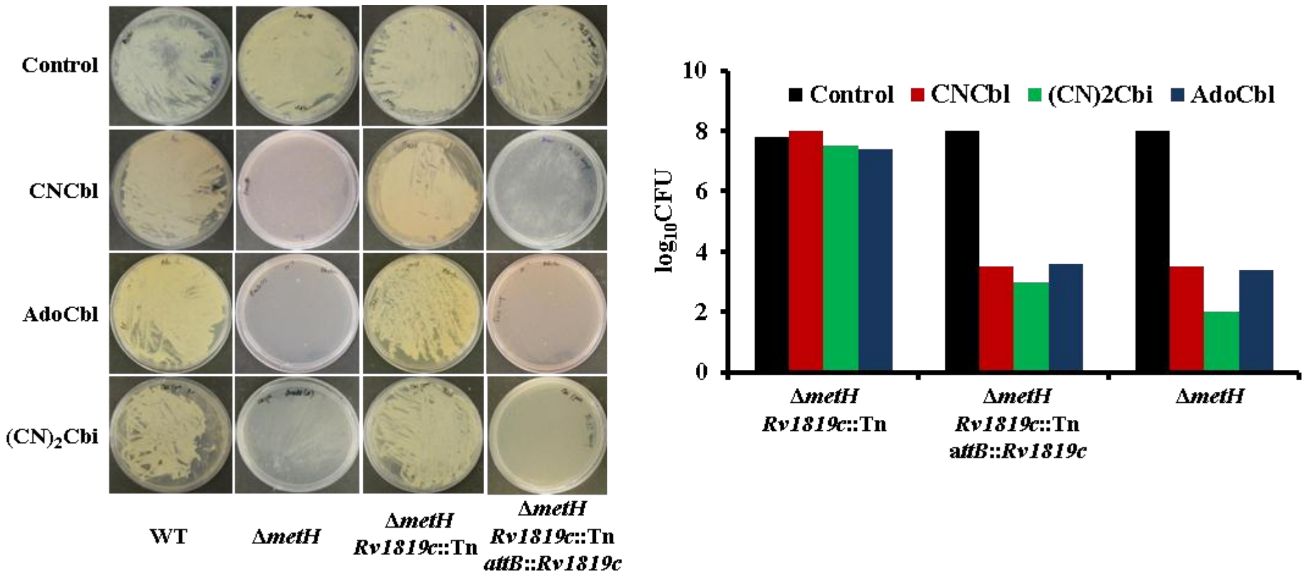


**Figure S5.** Disruption of *Rv1819c* alleviates sensitivity to corrinoids in *M. tuberculosis* Δ*metH*. Cells were incubated on solid 7H10 medium containing CNCbl or AdoCbl at 10 µg/ml, or $(CN)_2$Cbi at 1 µM, and CFUs scored after 4 weeks. Representative plates are shown from two independent experiments performed in duplicate, and CFU counts plotted in the adjacent bar graph.

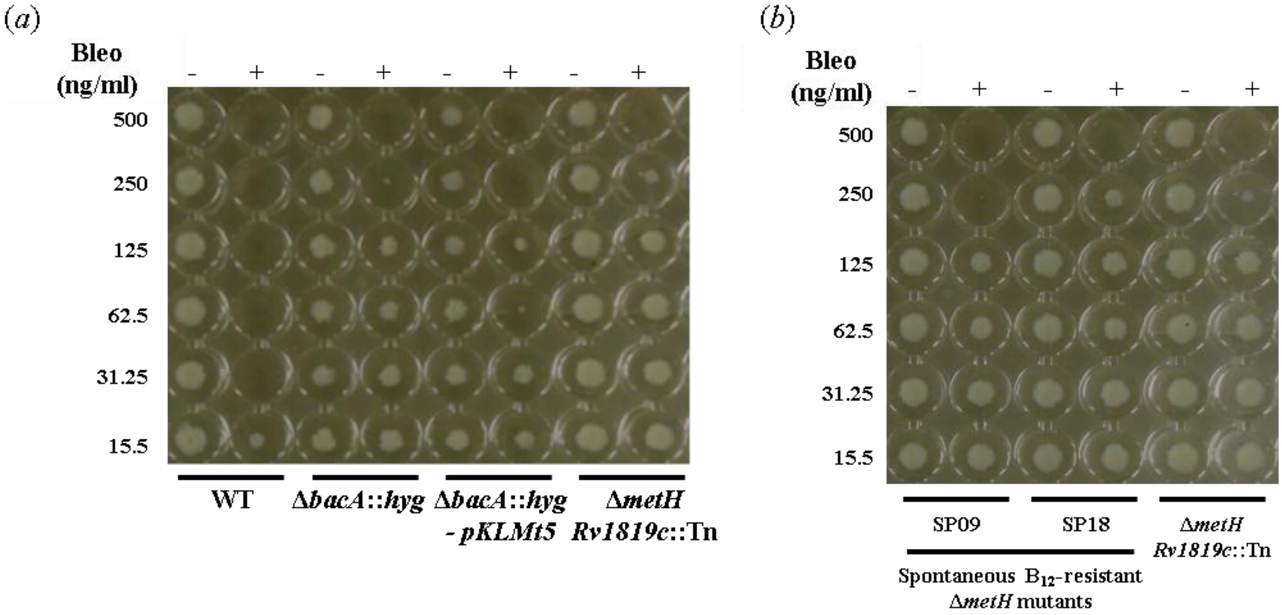


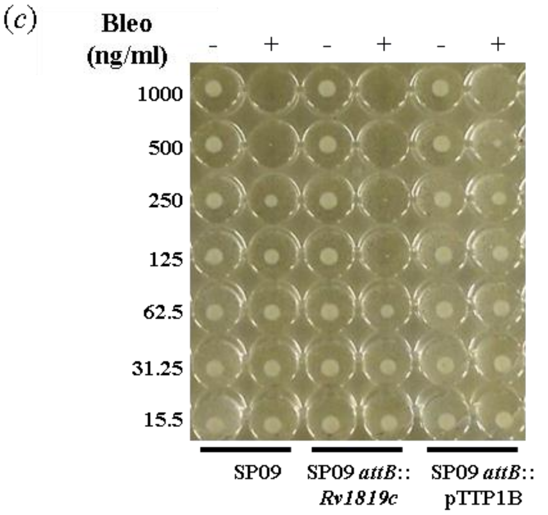


**Summary of Bleo susceptibilities**

| **Strain** | **MIC$_{99}$ (ng/ml)** |
|---|---|
| WT<br>Δ*metH*<br>Δ*prpDC* | 31.25 |
| SP09, SP18<br>SP09 *attB*::pTTP1B<br>Δ*metH Rv1819c*::Tn<br>Δ*bacA*::*hyg* | 500 |
| SP09 *attB*::*Rv1819c*<br>Δ*metH Rv1819c*::Tn *attB*::*Rv1819c*<br>Δ*bacA*::*hyg attB*::*Rv1819c* | 125 |

**Figure S6.** Inactivation of *Rv1819c* decreases the susceptibility of *M. tuberculosis* to bleomycin (Bleo). The broth microdilution method (4) was used to determine Bleo MIC values for (*a*) targeted (Δ*bacA*::*hyg*) and Tn insertion (Δ*metH Rv1819c*::Tn) mutants of *Rv1819c* and a complemented derivative (Δ*bacA*::*hyg pKLMt5)* (3) and (*b*) for spontaneous $B_{12}$-resistant mutants, SP09 and SP18 (Table S2), which contain SNPs in *Rv1819c*. (*c*) Complementation with full-length *Rv1819c* restores Bleo susceptibility in a spontaneous $B_{12}$-resistant mutant of Δ*metH*. The broth microdilution method (4) was used to determine Bleo MIC values for SP09, its complemented derivative (SP09 *attB*::*Rv1819c*), and a strain carrying the empty vector (SP09 *attB*::pTTP1B). The table summarizes the Bleo MIC$_{99}$ values for the *M. tuberculosis* strains tested in panels (*a*)-(*c*).

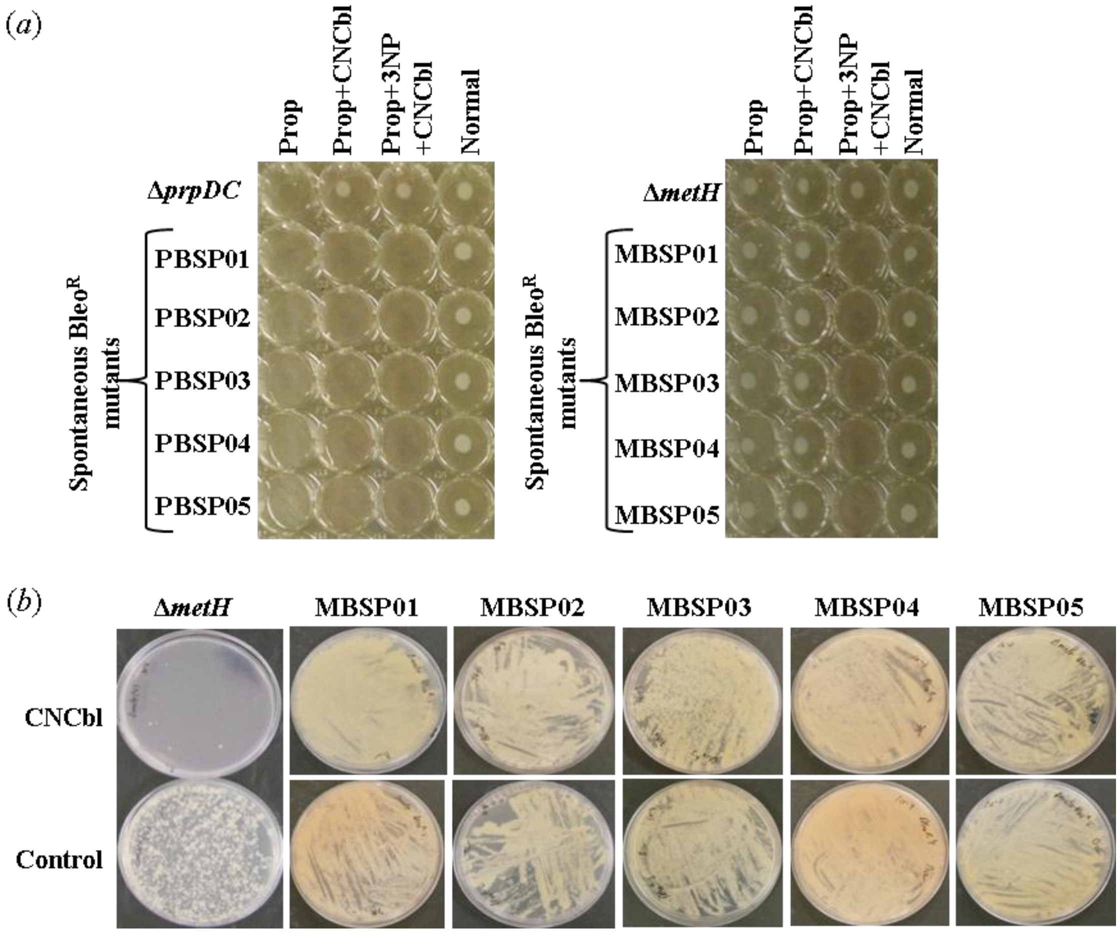


**Figure S7.** Overlapping functions of Rv1819c in $B_{12}$ transport and Bleo sensitivity. (*a*) Spontaneous Bleo$^R$ mutants containing SNPs in *Rv1819c* are impaired in their ability to utilize $B_{12}$ for propionate metabolism. (*b*) Cells were incubated on solid 7H10 medium containing 10 µg/ml CNCbl growth evaluated after 4 weeks. Representative plates are shown from two independent experiments performed in duplicate.

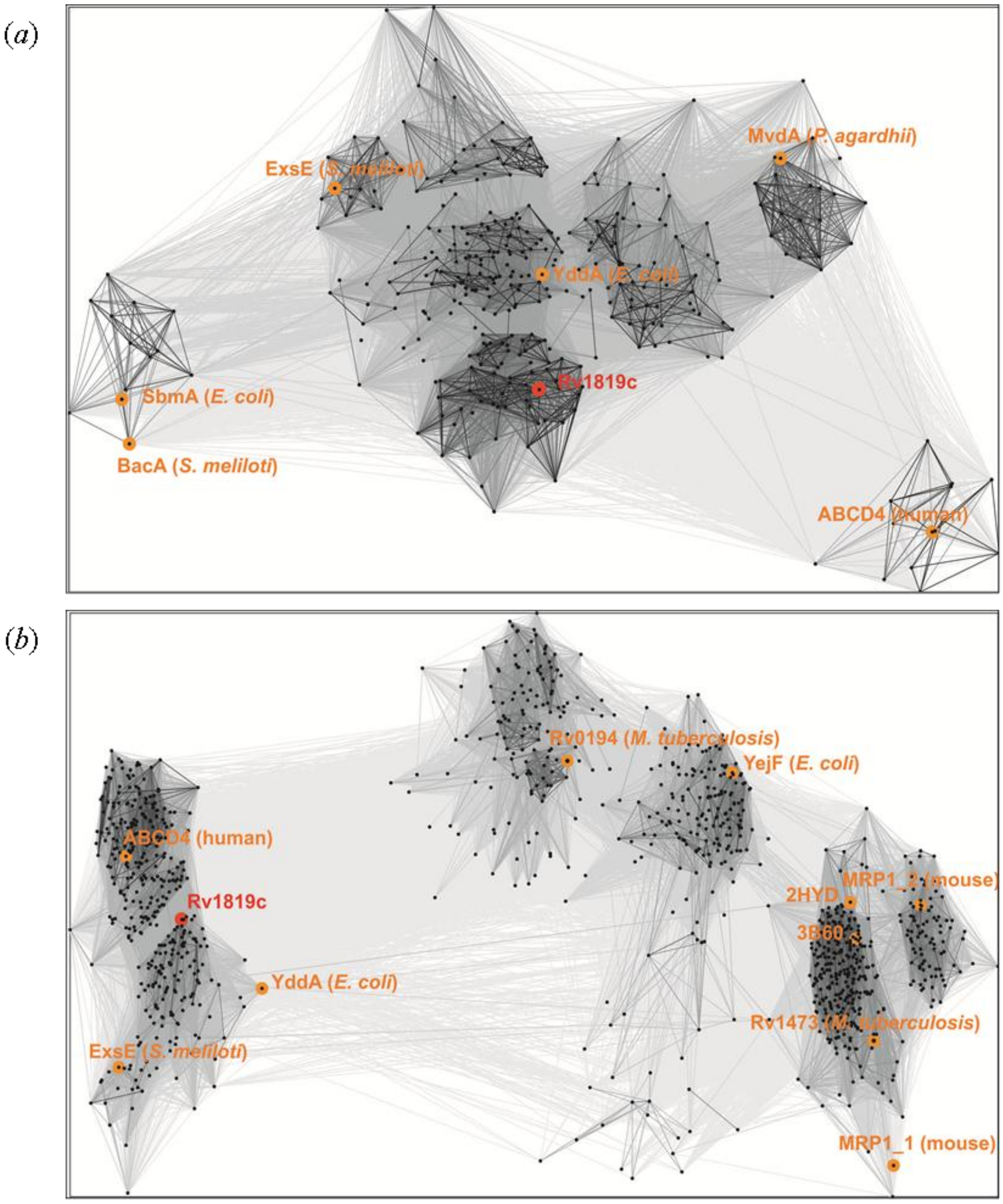


**Figure S8**. (*a*) The Rv1819c transmembrane domain (TMD) and its homologs were clustered using CLANS (Cluster Analysis of Sequences) (5). The analysis included 343 sequences, and each dot represents a single sequence. Only connections that correspond to P-values < 1e-10 are shown, with thicker lines indicating higher similarity. The Rv1819c TMD is denoted in red, with selected homologs (shown in orange) corresponding to the following Uniprot entries: Ydda (YDDA_ECOLI), ExsE (O54061_RHIML), SbmA (SBMA_ECOLI), BacA (BACA_RHIME), ABCD4 (ABCD4_HUMAN), and MvdA (B7SMU1_OSCAG). In (*b*), the Rv1819c nucleotide binding domain (NBD) and its homologs (total 1070 sequences) were clustered. Only connections corresponding to P-values < 1e-15 are shown. The red label indicates the Rv1819c NBD with selected homologs (orange) corresponding to the following Uniprot entries: ABCD4 (ABCD4_HUMAN), Ydda (YDDA_ECOLI), ExsE (O54061_RHIML), Rv0194 (O53645_MYCTU), Rv1473 (O53164_MYCTU), YejF (YEJF_ECOLI), and MRP1_1 and MRP1_2 – the NBD domains of MRP1 (MRP1_MOUSE). 2HYD and 3B60 denote the PDB codes of the crystal structures of *Staphylococcus aureus* Sav1866 and *Salmonella typhimurium* MsbA, respectively.

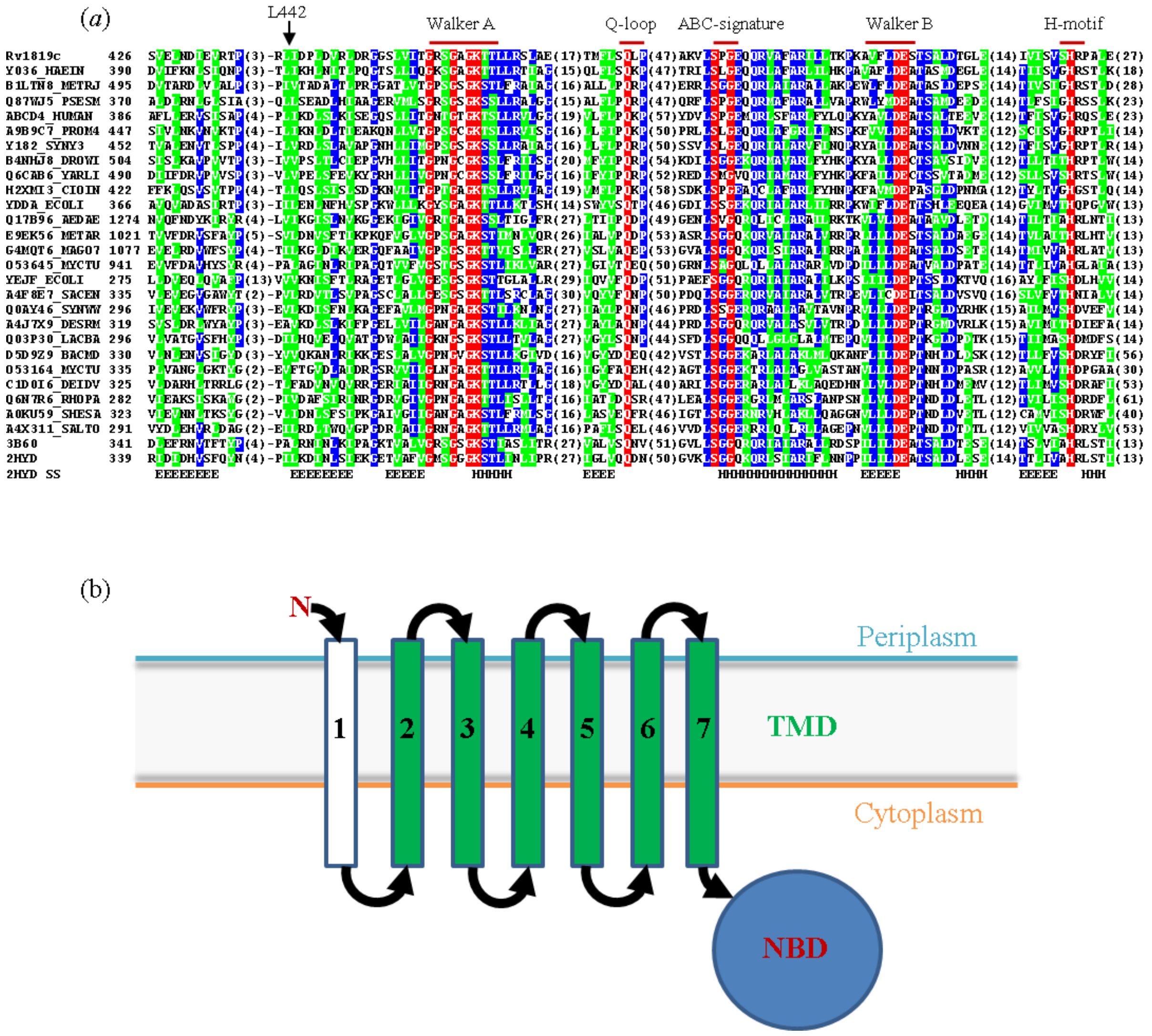


**Figure S9.** (*a*) Alignment of Rv1819c NBD with selected homologous sequences. Homologous sequences are denoted by their Uniprot accession numbers and structures used for modeling Rv1819c are indicated by their respective PDB codes. The secondary structure of 2HYD is shown below the alignment, where "E" indicates a β-strand and "H" indicates an α-helix. Conserved sequence motifs are shown above the alignment, and residues excluded from the alignment to make it more compact are indicated in parentheses. The arrow indicates the position of the L442 residue which is mutated in SP09 (Table S1). (*b*) Rv1819c topology diagram. Rv1819c is predicted to possess seven transmembrane helices. Six of these (colored green) correspond to transmembrane helices identified in *Staphylococcus aureus* Sav1866 (PDB ID: 2HYD) and *Salmonella typhimurium* MbsA (PDB ID: 3B60) and are included in the Rv1819c model.

## Table. S1. Strains, plasmids, PCR primers, oligonucleotides used in the study

| Strain | Description | Source |
|---|---|---|
| <u>*E. coli*</u> | | |
| DH5α | F- *φ80lacZΔM15* Δ(*lacZYA-argF*)U169 *recA1 endA1 hsdR17*(rk-, mk+) *phoAsupE44 thi*-1 *gyrA96 relA1* λ- | Invitrogen |
| DH5α λ(Pir) | λ pir lysogen of DH5α | Eric J. Rubin |
| <u>*Mtb*</u> | | |
| H37RvJO | Virulent reference laboratory strain; ATCC 25618 | Laboratory Stock |
| H37RvMA | Virulent reference laboratory strain from the laboratory of Dr. C. Sassetti; ATCC 27294 | Ref. 2 |
| Δ*metH*(BB) | *metH* deletion mutant of H37Rv lacking 1417 bp internal *Bgl*II-*Bcl*I fragment; $Hyg^R$ | Ref. 1 |
| Δ*metH* | *metH* deletion mutant of H37Rv; unmarked | This study |
| Δ*bacA*::*hyg* | *bacA* deletion mutant of H37Rv (Pasteur); lacking 541 bp internal *Eco*RV-*Xba*I fragment; $Hyg^R$ | Ref. 3 |
| Δ*bacA::hyg pKLMt5* | Complemened *bacA*::*hyg* mutant carrying 2283 bp region of the *M. tuberculosis* H37Rv chromosome containing the *bacA* gene flanked by 203 bp upstream and 161 bp downstream in the integrative vector pMV306K; $Hyg^R$ $Kan^R$ | Ref. 3 |
| Δ*bacA*::*hyg* *attB*::*Rv1819c* | Δ*bacA*::*hyg* complemented with full-length *Rv1819c* carried on pTTP1B vector integrated at *attB* locus; $Hyg^RGm^R$ | This study |
| Δ*metH Rv1819c*::Tn | Representative transposon mutants with disrupted *Rv1819c* identified in the $B_{12}$ uptake screen; $Kan^R$ | This study |
| Δ*metH Rv1314c*::Tn | Representative transposon mutants with disrupted *Rv1314c* (*pduO*) identified in the $B_{12}$ uptake screen; $Km^R$ | This study |
| Δ*metH Rv1819c*::Tn *attB*::*Rv1819c* | Δ*metH Rv1819c*::*Tn* complemented with full-length *Rv1819c* carried on pTTP1B vector integrated at *attB* locus; $Km^RGent^R$ | This study |
| Δ*prpDC* | *prpDC* deletion mutant of H37RvMA lacking the 2660 bp region from the start codon of *prpD* to the stop codon of *prpC* | This study |
| Δ*bacA*::*hyg* Δ*prpDC* | *prpDC* deletion mutant of Δ*bacA*::*hyg*; $Hyg^R$ | This study |
| Δ*bacA*::*hyg* Δ*prpDC* *attB::bacA* | Δ*bacA*::*hyg* Δ*prpDC* complemented with full-length *Rv1819c* carried on pTTP1B vector integrated at *attB* locus; $Hyg^R$ $Gent^R$ | This study |
| SP09 | Spontaneous vitamin $B_{12}$-resistant mutant of Δ*metH*(BB) | This study |
| SP18 | Spontaneous vitamin $B_{12}$-resistant mutant of Δ*metH*(BB) | This study |
| SP09 *attB*::*Rv1819c* | SP09 strain complemented with full-length *Rv1819c* carried on pTTP1B vector integrated at *attB* locus; $Hyg^RGent^R$ | This study |
| SP18 *attB*::*Rv1819c* | SP18 strain complemented with full-length *Rv1819c* carried on pTTP1B vector integrated at *attB* locus; $Hyg^RGent^R$ | This study |
| MBSP01, MBSP02 MBSP03, MBSP04 MBSP05 | Spontaneous bleomycin-resistant mutants of Δ*metH* | This study |
| MBSP01 *attB*::*Rv1819c* | MBSP01 complemented with full-length *Rv1819c* carried on pTTP1B vector integrated at *attB* locus; $Gent^R$ | This study |
| PBSP01, PBSP02 PBSP03, PBSP04 PBSP05 | Spontaneous bleomycin-resistant mutants of Δ*prpDC* | This study |
| PBSP04 *attB*::*Rv1819c* | PBSP04 complemented with full-length *Rv1819c* carried on pTTP1B vector integrated at *attB* locus; $Gent^R$ | This study |

| Plasmids | Description | Source |
|---|---|---|
| pAU100 | pJG1111 carrying Δ*prpDC* allele – fusion of 1kb PCR products upstream and downstream of *prpDC* eliminating 2660 bp *prpDC* coding sequence; $Km^R$ $Hyg^R$ | Ref. 6 |
| pTTP1B(Gm) | Gentamicin-resistant derivative of pTTP1B; $Gm^R$, $Amp^R$ | Ref. 7 |
| p*bacA*-Comp | pTTP1B(Gm) carrying 2274 bp region of the *M. tuberculosis* H37Rv chromosome containing the *Rv1819c* gene flanked by 203 bp upstream and 151 bp downstream sequence, $Gm^R$ | This study |

| Oligonucleotides | Sequence (5´-3´)[a] | Application | Amplicon properties/ region targeted/ reference |
|---|---|---|---|
| Linker 1 | TTTCTGCTCGAATTCAAGCTTCTAACGATGTACG GGGACACATG | Used to identify the Tn insertion site. PCR products obtained were sequenced using TnSeqF and TnSeqR | Ref. 8 |
| Linker 2 | TGTCCCCGTACATCGTTAGAACTACTCGTACCAT CCACAT | | |
| Y linker | CTGCTCGAATTCAAGCTTCT | | |
| TnSeqF | CGAGATAGGGTTGAGTGT | Sequencing primer | Sequencing primers used to identify Tn insertion sites from the plasmids obtained by rescue cloning |
| TnSeqR | GTTGGCTACCCGTGATATTG | Sequencing primer | |
| prpDC-F | *GGGGG*CTGCTCTGCGGCACGGTG | Forward primer used for PCR-based genotyping of the *prpDC* and Δ*prpDC* alleles | 1073 bp amplicon generated from wild-type *prpDC* allele using prpDC-F and prpDC-R |
| prpDC-R | *GGGGG*ATCTTGTAGGCCATGTGCTC | Reverse primer used for PCR genotyping of *prpDC* alleles | |
| prpDC-R2 | *GGGGG*TACAACAGGATCTTGGCGAC | Reverse primer used for PCR genotyping of Δ*prpDC* allele | 1195bp amplicon from Δ*prpDC* using prpDC-F and PrpDC-R2 |
| Tn_Mar_In | CGCACTGAGAAGCCCTTA | Forward and reverse primer used for PCR-based screen to confirm the presence of MycoMar-1 sequence in Tn mutants | 838-bp amplicon generated from Tn mutants |
| Tn_Mar_Out | GCAAGGTGAGATGACAGGA | | |
| *met*B12seqF1 | GGCCCAGTAGCCTTCGGT | Sequencing primer | *met*B12seqF1& R2 were used to amplify the genomic region of the $B_{12}$ riboswitch located upstream of *metE.* The resulting 500 bp amplicon was sequenced using the same primers |
| *met*B12seqR1 | GGCCAGTAGGAGCACCCA | Sequencing primer | |
| *met*B12seqF2 | GGCTGGCAGGTCTTCGGA | Sequencing primer | |
| *met*B12seqR2 | GGCCGATGTCACCGGAGT | Sequencing primer | |
| bacA F1 | CGTGACCACAAATGACAT | Sequencing primer | bacA F1& R5 were used to amply the genomic region of *Rv1819c* with 200 bp upstream and downstream flaking sequences. The resulting 2264 bp amplicon was sequenced using the indicated primers. |
| bacA F2 | TGGCTGATGCTCGGCGTG | Sequencing primer | |
| bacA F3 | ATCGGGCGGCCCCTGATC | Sequencing primer | |
| bacA F4 | ATCATCCGATTGCATGGG | Sequencing primer | |
| bacA F5 | TGACCGGCTGGACGAGGA | Sequencing primer | |
| bacA R1 | AGATGGAAAACAGGTGGC | Sequencing primer | |
| bacA R2 | ATCCTCGCACGCCTGAGC | Sequencing primer | |
| bacA R3 | ACGGTCCAGAACATTGCG | Sequencing primer | |
| bacA R4 | GAGTCGTGAATGTTGCCG | Sequencing primer | |
| bacA R5 | CGCCACCTTGGTCAGCGT | Sequencing primer | |
| bacA Comp_F | G*GGCG***GAATTC**GGCAGCCGTC | Primers used to amplify *Rv1819c* with flanking region for construction of p*bacA*-Comp | bacA Comp_F and bacA Comp_R were used to generate the 2274 bp complementing allele containing *Eco*RI restriction sites |
| bacA Comp_R | *GGCGG***GAATTC**GTGACCACAA | Primers used to amplify *Rv1819c* with flanking region for construction of p*bacA*-Comp | |
| TTP1b | GTCACCGAAAGGCGTGCCCTTGTC | TTP1b and bacA Compl_Forward primer used for PCR screen to confirm the transformants | |

a. GC-clamp sequences (non-H37Rv) are italicized; Restriction sites are shown in bold

**Table S2:** ***Rv1819c*** **polymorphisms in spontaneous mutants of Δ*metH* and Δ*prpDC***

| Parent | Strain ID | Selected on | SNP | Amino acid change[§] | Complemented by full-length *Rv1819c*[†] | Whole-genome sequence data[‡] |
|---|---|---|---|---|---|---|
| **Δ*metH* (BB)** | **Parental** | - | - | - | - | **Coverage: 83.5x Additional polymorphisms: none** |
| **Δ*metH* (BB)** | **SP09** | **$B_{12}$** | **T – C (1325)** | **L442S** | **Yes** | **Coverage: 121.4x Additional polymorphisms: none** |
| **Δ*metH* (BB)** | **SP18** | **$B_{12}$** | **C – T (1045)** | **P349T** | **Yes** | - |
| **Δ*metH* (BB)** | **SP06** | **$B_{12}$** | **G – A (459)** | **W153*** | **ND** | - |
| **Δ*metH* (BB)** | **SP03** | **$B_{12}$** | **C – T (1027)** | **Q343*** | **ND** | - |
| **Δ*metH*** | **Parental** | - | - | - | - | - |
| **Δ*metH*** | **MBSP01** | **Bleomycin** | **G – A (1232)** | **G411D** | **ND** | - |
| **Δ*metH*** | **MBSP04** | **Bleomycin** | **G – A (35)** | **W12*** | **ND** | - |
| **Δ*metH*** | **MBSP05** | **Bleomycin** | | | **ND** | - |
| **Δ*prpDC*** | **Parental** | - | - | - | - | - |
| **Δ*prpDC*** | **PBSP01** | **Bleomycin** | **G – A (347)** | **W52*** | **ND** | - |
| **Δ*prpDC*** | **PBSP02** | **Bleomycin** | | | **ND** | - |
| **Δ*prpDC*** | **PBSP03** | **Bleomycin** | | | **ND** | - |
| **Δ*prpDC*** | **PBSP04** | **Bleomycin** | | | **Yes** | - |

[§] An asterisk(*) denotes a nonsense mutation
[†] ND, not done
[‡] Polymorphisms were determined relative to the parental strain, H37RvJO (Ref. 2)

**Supplementary References**

1. Warner DF, Savvi S, Mizrahi V, Dawes SS. 2007 A riboswitch regulates expression of the coenzyme $B_{12}$-independent methionine synthase in *Mycobacterium tuberculosis*: implications for differential methionine synthase function in strains H37Rv and CDC1551. *J Bacteriol*. **189**, 3655-3659. (doi:10.1128/JB.00040-07)

2. Ioerger TR, Feng Y, Ganesula K, Chen X, Dobos KM, Fortune S, Jacobs WR, Jr., Mizrahi V, Parish T, Rubin E, Sassetti C, Sacchettini JC. 2010 Variation among genome sequences of H37Rv strains of *Mycobacterium tuberculosis* from multiple laboratories. *J Bacteriol*. **192**, 3645-3653. (doi:10.1128/JB.00166-10)

3. Domenech P, Kobayashi H, LeVier K, Walker GC, Barry CE, 3rd. 2009 BacA, an ABC transporter involved in maintenance of chronic murine infections with *Mycobacterium tuberculosis*. *J Bacteriol*. **191**, 477-485. (doi:10.1128/JB.01132-08)

4. Ward A, Reyes CL, Yu J, Roth CB, Chang G. 2007 Flexibility in the ABC transporter MsbA: Alternating access with a twist. *Proc Natl Acad Sci U S A*. **104**, 19005-19010. (doi:10.1073/pnas.0709388104)

5. Frickey T, Lupas A. 2004 CLANS: a Java application for visualizing protein families based on pairwise similarity. *Bioinformatics*. **20**, 3702-3704. (doi:10.1093/bioinformatics/bth444)

6. Muñoz-Elías EJ, Upton AM, Cherian J, McKinney JD. 2006 Role of the methylcitrate cycle in *Mycobacterium tuberculosis* metabolism, intracellular growth, and virulence. *Mol Microbiol*. **60**, 1109-1122. (doi:10.1111/j.1365-2958.2006.05155.x)

7. Abrahams GL, Kumar A, Savvi S, Hung AW, Wen S, Abell C, Barry CE, 3rd, Sherman DR, Boshoff HI, Mizrahi V. 2012 Pathway-selective sensitization of *Mycobacterium tuberculosis* for target-based whole-cell screening. *Chem Biol*. **19**, 844-854. (doi:10.1016/j.chembiol.2012.05.020)

8. Kwon YM, Ricke SC. 2000 Efficient amplification of multiple transposon-flanking sequences. *J Microbiol Methods*. **41**, 195-199. (doi:10.1016/S0167-7012(00)00159-7)
